# The primary ossification of the human fetal ischium: CT, digital-image analysis, and statistics

**DOI:** 10.1007/s00276-018-2171-5

**Published:** 2018-12-20

**Authors:** Mariusz Baumgart, Marcin Wiśniewski, Magdalena Grzonkowska, Mateusz Badura, Michał Szpinda, Katarzyna Pawlak-Osińska

**Affiliations:** 1 Department of Normal Anatomy, The Ludwik Rydygier Collegium Medicum in Bydgoszcz, The Nicolaus Copernicus University in Toruń, Łukasiewicza 1 Street, 85-821 Bydgoszcz, Poland; 2 Department of Otolaryngology and Oncology, The Ludwik Rydygier Collegium Medicum in Bydgoszcz, The Nicolaus Copernicus University in Toruń, Bydgoszcz, Poland

**Keywords:** Ischium, Bone development, Osteogenesis, Fetal development

## Abstract

**Purposes:**

Details concerning the normal growth of the pelvic girdle in the fetus are of importance in the early detection of congenital defects. This study was executed to quantitatively evaluate the primary ossification center of the ischium with relation to its linear, planar and volumetric parameters.

**Materials and methods:**

Using methods of CT, digital-image analysis, and statistics, geometrical dimensions of the ischium’s primary ossification center in 42 spontaneously aborted human fetuses (21 ♂ and 21 ♀) aged 18–30 weeks were calculated.

**Results:**

With no sex and laterality differences, the best fit growth dynamics for the ischium’s primary ossification center were displayed by the following functions: *y* = − 10.045 + 0.742 × age ± 0.013 (*R*^2^ = 0.97) for its vertical diameter, *y* = − 5.212 + 0.385 × age ± 0.008 (*R*^2^ = 0.97) for its sagittal diameter, *y* = − 36.401 + 0.122 × (age)^2^ ± 45.534 (*R*^2^ = 0.96) for its projection surface area, and *y* = − 1052.840 + 368.470 × ln(age) ± 12.705 (*R*^2^ = 0.91) for its volume.

**Conclusions:**

Neither male–female nor right–left differences are found for any of the morphometric parameters of the ischium’s primary ossification center. With relation to fetal ages in weeks, the ischium’s primary ossification center grows proportionately in vertical and sagittal diameters, second-degree polynomially in projection surface area, and logarithmically in volume. The quantitative findings of the ischium’s primary ossification center are considered age-specific reference data of relevance in the diagnostics of innate defects.

## Introduction

Until puberty, the coxal bone consists of three parts: the ilium, ischium, and pubis, which are separated from each other by cartilage. As late as at the age of 16–18 years, these three bones fuse onto one coxal bone and contribute to a socket of the hip joint, i.e., the acetabulum. From a clinical point of view, the development of the hip joint presents a common interest in sports medicine, manual therapy, biomechanics, and anthropology [10, 12].

Among the three constituents of the coxal bone, the ilium is the first to ossify at week 9 of gestation [9, 10]. The ossification process of the ischium and pubis commences as late as at weeks 18 and 20, respectively [10, 18]. In relation with the acetabulum, the primary ossification center of the ischium is somewhat backwards and downwards in position [15]. Although the fusion of primary ossification centers of three components of the pelvic bone initially refers to the ischium and pubis [19], at birth, the ischium is still separated from the pubis by the ischiopubic synchondrosis [14]. The age at which this synchondrosis tends to ossify occurs as late as 4–12 years [14]. Such an ossification process is initiated by the fusion of the ischial ramus and the inferior pubic ramus to form the ischiopubic ramus, while the fusion within the acetabulum starts during puberty [18]. The secondary ossification centers of the pelvic bone refer to the iliac crest, anterior inferior iliac spine, ischial spine and ischial tuberosity, and ossify during puberty and adulthood [12].

As hypothesized by Verbruggen et al. [18], towards the end of month 6 of gestation, the ischium is easily identified due to its comma-shaped appearance. Thus, its upper sagittal dimension is greater than its lower sagittal dimension, and besides its ramus is directed forwards. Of note, the upper, lower, and posterior edges of the ischium are convex, while the anterior edge is concave. Inasmuch as the pelvic surface of the ischium is smooth, its dorsal surface has a conspicuous cavity that contributes to the acetabulum. At birth, the articular part of acetabulum is located on the posterior-lateral surface of the ischium, while the non-articular part is located anteriorly.

A clear understanding of the prenatal growth of ischium may be conducive in the early detection of some congenital faults concerning the pelvis and lower limbs [4, 6, 12], and, therefore, is critical in anatomy, pediatrics, neonatology, gynecology and obstetrics.

Although the timing of ossification of each constituent of the coxal bone has accurately been identified [9, 10, 13, 15, 18], to date, no morphometric measurements of the ischium’s primary ossification centers have been reported. Our study may provide numerous pieces of information valuable for the early diagnostics of normal and abnormal development of the skeletal system in human fetuses. The present study is the first to quantitatively assess the ischium’s primary ossification center in human fetuses with the use of advanced imaging methods.

The purposes of this study were:

- to perform morphometric analysis of the ischium’s primary ossification center in human fetuses with relation to its linear, superficial and spatial parameters to determine their normative values;
- to establish possible sex and laterality differences for all analyzed parameters;
- to compute growth dynamics for all analyzed parameters, expressed by best-matched mathematical models.

## Materials and methods

The sample population consisted of 42 human fetuses (21 males and 21 females) aged 18–30 weeks, originating from spontaneous miscarriages or preterm deliveries. Prior to the year 2000, the retention of fetal material was approved by the Bioethics Committee of Collegium Medicum of the Nicolaus Copernicus University (KB 275/2011). The inclusion of the fetuses studied was grounded in the assessment of their external morphology and statistical cards with the course of pregnancy. Since on macroscopic examination, no explicit conspicuous morphological malformations were found, all included specimens were considered normal. The fetal ages were determined according to the crown-rump length (CRL). Table 1 lists the characteristics of the study group, comprising ages in weeks, number, and sex of the fetuses.

**Table 1:** Age, number, and sex of the fetuses studied

Age (weeks)	Crown-rump length (mm)				Number of fetuses	Sex	
	Mean	SD	Min.	Max.		♂	♀
18	133.3	5.80	130.0	140.0	3	1	2
19	150.00	3.03	146.0	154.0	6	2	4
20	159.67	0.58	159.0	160.0	3	2	1
21	174.67	3.51	171.0	178.0	3	2	1
22	184.50	2.12	183.00	186.00	2	0	2
23	196.33	1.15	195.0	197.0	3	1	2
24	208.67	3.81	204.0	213.0	9	5	4
25	214.00	–	214.0	214.0	1	0	1
26	229.00	5.70	225.0	233.0	2	1	1
27	239.25	2.36	236.0	241.0	4	4	0
28	249.50	0.70	249.0	250.0	2	0	2
29	253.00	–	253.0	253.0	1	0	1
30	263.67	1.15	263.0	265.0	3	3	0
Total					42	21	21

**Fig. 1:**
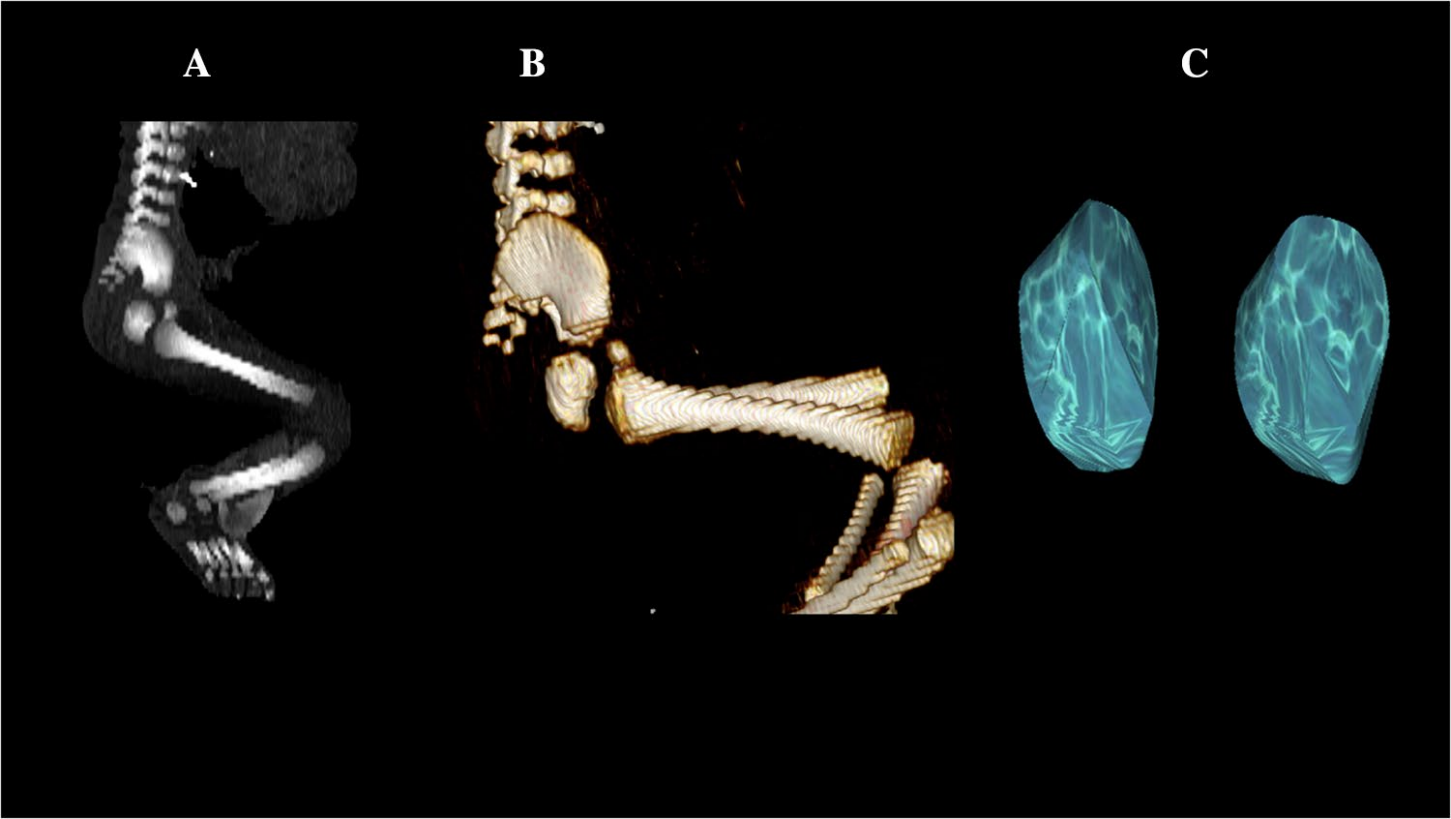
CT of a male human fetus aged 26 weeks in the sagittal projection (**a**), 3D reconstruction of the pelvis in the sagittal projection (**b**), and primary ossification center of the ischium (**c**) using Osirix 3.9

Using the Siemens-Biograph 128 mCT scanner (Siemens Healthcare GmbH, Erlangen, Germany) located at the Department of Positron Emission Tomography and Molecular Imaging (Oncology Center, Collegium Medicum of the Nicolaus Copernicus University, Bydgoszcz, Poland), scans of fetuses in DICOM formats were acquired at 0.4 mm intervals (Fig. 1). The gray scale of achieved CT pictures expressed in Hounsfield units (HU) varied from − 275 to − 134 for a minimum and from + 1165 to + 1558 for a maximum. Therefore, the window width (WW) altered from 1.404 to 1.692, and the window level (WL) varied from + 463 to + 712. The specifics of the imaging protocol were presented by the following: mAs—60, kV—80, pitch—0.35, FoV—180, rot. time—0.5 s, while the specifics of CT data were: slice thickness—0.4 mm, image increment—0.6 mm, and kernel—B45 f-medium. In spite of the cartilaginous stage of the coxal bone, the ischium’s primary ossification centers were already clearly visible and contoured [5, 11]. This was a prerequisite to perform their morphometric analysis regarding linear, planar, and volumetric dimensions.

**Fig. 2:**
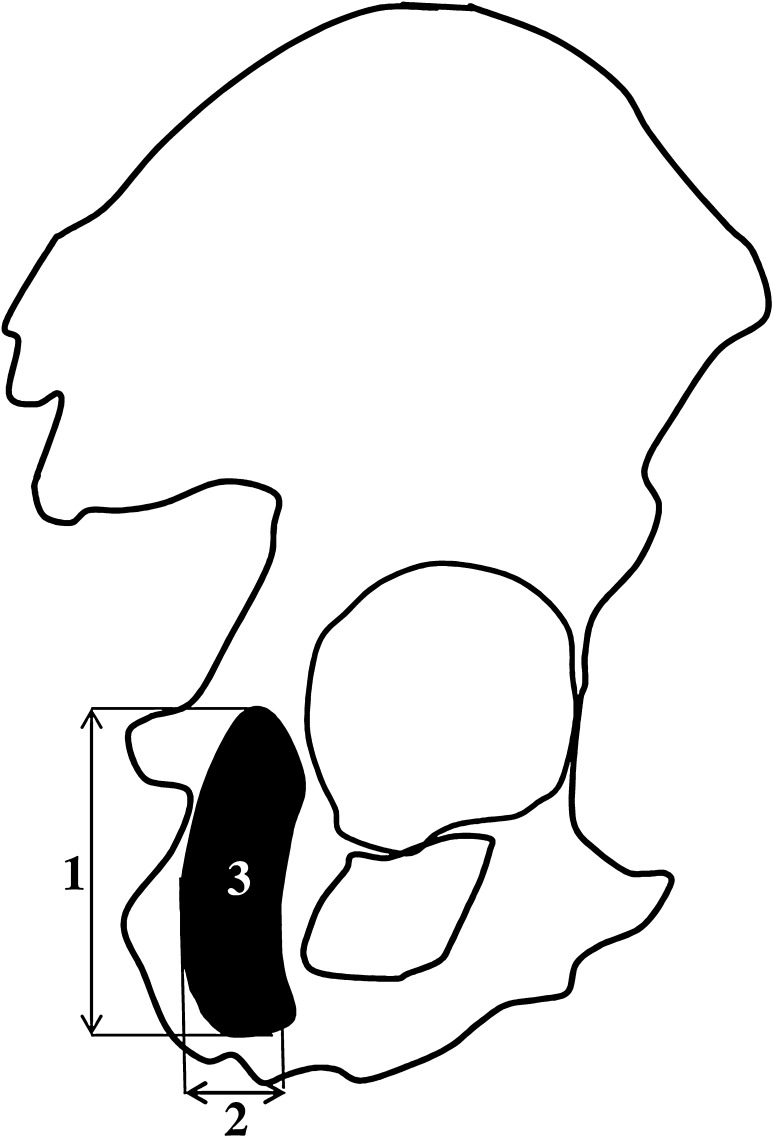
Measurement scheme of the ischium’s primary ossification center in the sagittal plane. 1—vertical diameter, 2—sagittal diameter, and 3—projection surface area

The following four measurements of the ischium’s primary ossification center were conducted:

1. vertical diameter, based on the determined distance between the upper and lower borderlines of the ossification center in the sagittal plane (Fig. 2);
2. sagittal diameter, based on the determined distance between the anterior/ventral and posterior/dorsal borderlines of the ossification center in the sagittal plane (Fig. 2);
3. projection surface area, based on the determined contour of the ossification center in the sagittal plane (Fig. 2);
4. volume, calculated using advanced diagnostic imaging tools for 3D reconstruction, taking into account position and the absorption of radiation by bone tissue (Fig. 1c).

The numerical data was statistically analyzed. Distribution of variables was checked using the Shapiro–Wilk (*W*) test, while homogeneity of variance was checked using Fisher’s test. The results were expressed as arithmetic means ± standard deviations (SD). To compare the means, Student’s *t* test for independent variables and one-way analysis of variance were used, while Tukey’s test was used for post-hoc analysis. If no similarity of variance occurred, the non-parametric Kruskal–Wallis test was used. The characterization of developmental dynamics of the analyzed parameters was based on linear and non-linear regression analysis. The match between the estimated functions and numerical findings was evaluated based on the coefficient of determination (*R*^2^).

## Results

Tables 2 and 3 present arithmetic means and standard deviations of vertical and sagittal diameters, projection surface area, and volume of the right and left ischium’s primary ossification centers in human fetuses at the examined age range. Of note, the statistical analysis exposed neither significant sex nor bilateral differences, thus allowing us to compute only one growth curve for each analyzed parameter.

**Table 2:** Vertical and sagittal diameters, projection surface area, and volume of the right ischium’s ossification center

Age (weeks)	Number of fetuses	Ossification centers of the right ischium							
		Vertical diameter (mm)		Sagittal diameter (mm)		Projection surface area (mm^2^)		Volume (mm^3^)	
		Mean	SD	Mean	SD	Mean	SD	Mean	SD
18	3	3.15	0.20	1.64	0.10	5.41	0.66	7.53	2.14
19	6	3.61	0.25	1.88	0.13	7.03	0.96	13.39	2.47
20	3	4.77	0.61	2.48	0.31	12.14	2.87	29.01	4.42
21	3	6.15	0.35	3.20	0.18	19.93	2.24	71.36	33.52
22	2	6.82	0.11	3.55	0.06	24.41	0.80	109.09	7.34
23	3	7.40	0.12	3.85	0.06	28.67	0.88	121.05	3.42
24	9	7.46	0.16	3.88	0.09	29.17	1.38	135.48	1.95
25	1	7.81	–	4.06	–	31.90	–	138.47	–
26	2	8.54	0.01	4.46	0.03	38.11	0.13	139.72	0.54
27	4	9.90	0.28	5.15	0.14	51.12	2.86	150.79	7.46
28	2	10.62	0.14	5.52	0.07	58.83	1.56	166.84	0.54
29	1	11.03	–	5.74	–	63.44	–	168.90	–
30	3	12.17	0.15	6.33	0.08	77.16	1.85	171.22	1.00

**Table 3:** Vertical and sagittal diameters, projection surface area, and volume of the left ischium’s ossification center

Age (weeks)	Number of fetuses	Ossification centers of the left ischium							
		Vertical diameter (mm)		Sagittal diameter (mm)		Projection surface area (mm^2^)		Volume (mm^3^)	
		Mean	SD	Mean	SD	Mean	SD	Mean	SD
18	3	3.14	0.14	1.62	0.10	5.42	0.66	7.54	2.12
19	6	3.60	0.23	1.89	0.14	7.05	0.96	13.32	2.37
20	3	4.85	0.69	2.40	0.27	12.25	2.94	28.30	3.86
21	3	6.11	0.37	3.22	0.18	19.96	2.25	72.05	35.13
22	2	6.82	0.08	3.51	0.15	24.44	0.82	109.54	8.76
23	3	7.39	0.09	3.88	0.11	28.74	0.92	121.51	2.73
24	9	7.53	0.11	3.88	0.09	29.10	1.29	135.50	1.88
25	1	7.84	–	4.02	–	31.92	–	138.48	–
26	2	8.60	0.01	4.40	0.02	38.08	0.06	140.97	0.18
27	4	9.95	0.28	5.13	0.15	51.14	2.69	151.80	7.41
28	2	10.61	0.18	5.48	0.06	58.79	1.76	166.93	1.82
29	1	11.08	–	5.83	–	63.44	–	169.46	–
30	3	12.29	0.09	6.33	0.06	77.78	2.04	172.52	1.02

**Fig. 3:**
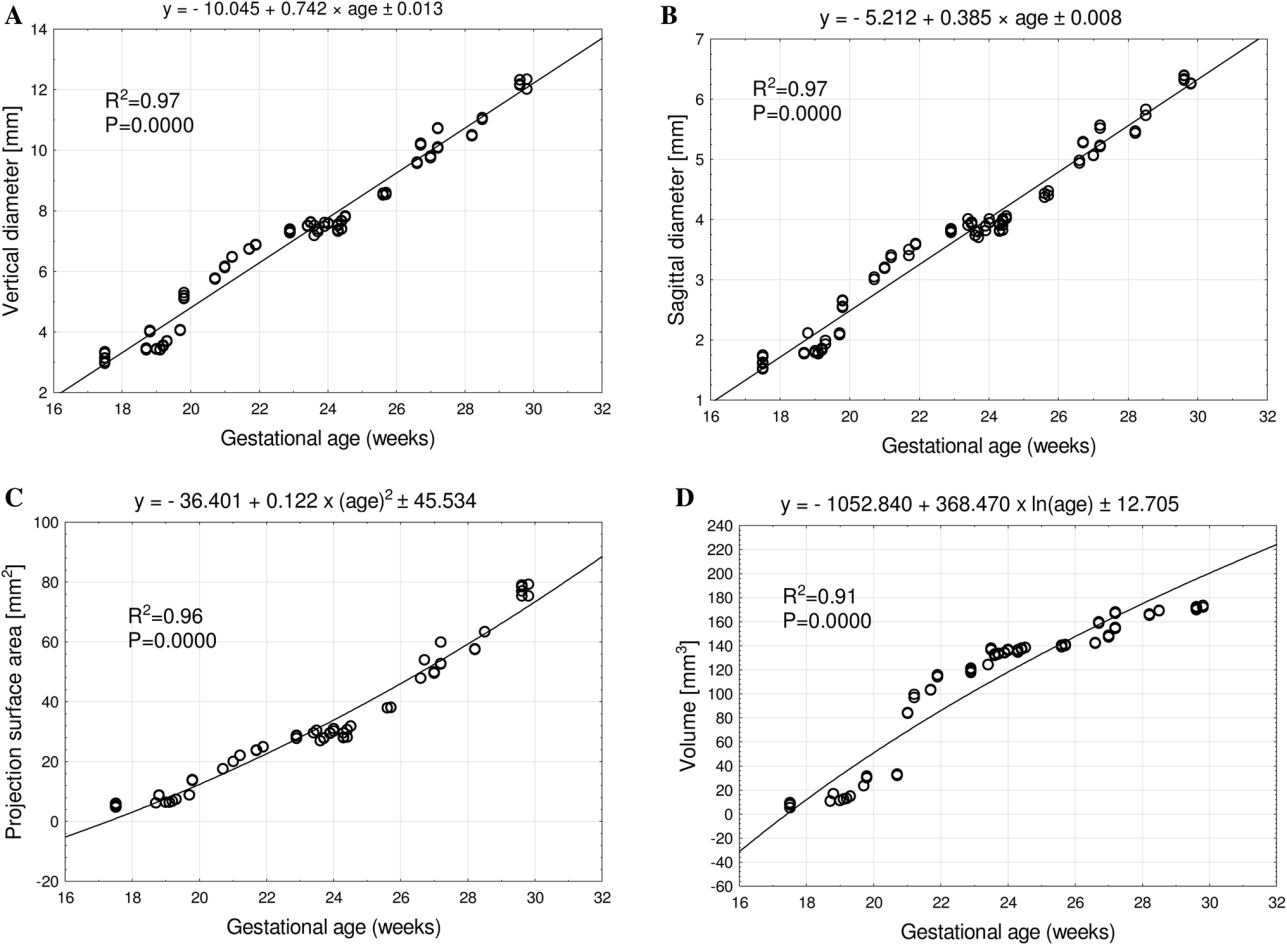
Regression lines for vertical diameter (**a**), sagittal diameter (**b**), projection surface area (**c**), and volume (**d**) of the ischium’s primary ossification center

The mean vertical diameter of the right ischium’s primary ossification center in fetuses at 18–30 weeks increased from 3.15 ± 0.20 to 12.17 ± 0.15 mm, while that of the left ischium’s primary ossification center increased from 3.14 ± 0.14 to 12.29 ± 0.09 mm, according to the linear function: *y* = − 10.045 + 0.742 × age ± 0.013 (*R*^2^ = 0.97)—(Fig. 3a).

The mean sagittal diameter of the right ischium’s primary ossification center ranged from 1.64 ± 0.10 mm at week 18 to 6.33 ± 0.08 mm at week 30, while that of the left ischium’s primary ossification center ranged from 1.62 ± 0.11 mm at 18 weeks to 6.33 ± 0.06 mm at 30 weeks, following the linear function: *y* = − 5.212 + 0.385 × age ± 0.008 (*R*^2^ = 0.97)—(Fig. 3b).

The mean projection surface area of the ischium’s primary ossification center at fetal agesof 18–30 weeks ranged from 5.41 ± 0.66 to 77.16 ± 1.85 mm^2^ on the right, and from 5.42 ± 0.66 to 77.78 ± 2.04 mm^2^ on the left, in accordance with the quadratic function: *y* = − 36.401 + 0.122 × (age)^2^ ± 45.534 (*R*^2^ = 0.96)—(Fig. 3c).

During that time, the mean volume of the right and left ischium’s primary ossification centers ranged from 7.53 ± 2.14 to 171.22 ± 1.00 mm^3^, and from 7.54 ± 2.12 to 172.52 ± 1.02 mm^3^, respectively, following the natural logarithmic function: *y* = − 1052.840 + 368.470 × ln(age) ± 12.705 (*R*^2^ = 0.91)—(Fig. 3d).

## Discussion

Francis [13] obtained 640 radiographs of fetuses with CRL values ranging from 32 to 472 mm. By examining primary ossification centers of the pelvis in male and female fetuses with CRL less than 160 mm, no distinct sex difference in their timing was observed. Contrariwise, in fetuses with CRL greater than 160 mm, primary ossification centers in female fetuses could be noticed earlier than those in males. The primary ossification center of ischium appeared between the greater sciatic notch and the wing of ilium, and was present in one male fetus with CRL of 112 mm. In the group of fetuses with CRL between 81 and 135 mm, the ischium’s primary ossification center occurred in 36% of cases, while in the fetuses with CRL greater than 135 mm, the ossification center was present in all individuals. Neither sex nor laterality differences regarding the timing of the ischium’s primary ossification centers were found. Equally, in our study, the ischium’s primary ossification center did not demonstrate any sex and laterality differences. These findings remain in line with studies conducted using CT for primary ossification centers of the femur [3] and ilium [2] in human fetuses.

This paper is the first report with the use of CT and digital-image analysis to precisely analyze the ischium’s ossification center in human fetuses and to compute its growth dynamics. The ischium’s primary ossification center grew proportionately to age in respect to its vertical and sagittal diameters, as follows: *y* = − 10.045 + 0.742 × age ± 0.013 and *y* = − 5.212 + 0.385 × age ± 0.008, respectively. Furthermore, its projection surface area followed the quadratic function: *y* = − 36.401 + 0.122 × (age)^2^ ± 45.534, while its volume increased following the natural logarithmic function: *y* = − 1052.840 + 368.470 × ln(age) ± 12.705. In our study about the development of the ilium’s primary ossification center in fetuses aged 18–30 weeks, its growth dynamics regarding the vertical and sagittal diameters followed the natural logarithmic functions: *y* = − 63.138 + 33.413 × ln(CRL) ± 1.609 and *y* = − 59.220 + 31.353 × ln(CRL) ± 1.736, respectively. In turn, the projection surface area and volume of the ossification center increased linearly: *y* = − 105.681 + 1.137 × CRL ± 16.035 and *y* = − 478.588 + 4.035 × CRL ± 14.332, respectively [2].

In the professional literature, we failed to find any reports concerning the dimensions of the ischium’s primary ossification center, which evidently precludes a more comprehensive discussion on this topic.

Both the numerical data and growth dynamics of the ischium’s primary ossification center obtained in the present study are considered age-specific references that may be useful in diagnosing skeletal dysplasias, characterized by a disrupted or restricted fetal growth, and exemplified by cleidocranial dysplasia, ischiovertebral dysplasia, arrested or abnormal pelvic development, hip dislocation, and campomelic dysplasia. Skeletodysplasias may result in aggravated and rapidly advancing scolioses associated with abnormalities in the pelvic bone. One of the disorders associated with kyphoscoliosis is cleidocranial dysplasia, which also involves abnormalities in cranial ossification, hypoplasia or dysplasia of the clavicle, delayed ossification of the pelvic bones, and abnormal ossification of bony shafts [8]. Abnormalities of pelvic ossification with concomitant kyphoscoliosis are also typical of ischiovertebral dysplasia. In cleidocranial dysplasia, delayed ossification more often affects the pubis, while in ischiovertebral dysplasia—the ischium; the degree of scoliosis is milder in the former disorder [8]. Arrested or abnormal pelvic development, as well as some growth disturbances in female fetuses can cause changes in the pelvis that usually occur as a pelvic narrowing of a lateral, oblique, or heart-shaped type, which consequently is of key importance at birth [12]. Furthermore, vertical positioning of the ischium occurs in hip dislocation and campomelic dysplasia (CMD), which may already be observed at an early stage of ischium ossification by detecting ossification only of the ramus of ischium [12]. If skeletal dysplasia is suspected, using ultrasound alone is not sufficient to make a comprehensive diagnosis. In such cases, a radiographic examination [12], ultrasound imaging [17], CT [2, 3, 20], and MRI [16] should be employed.

Van Zalen-Sprock et al. [17] compared the sensitivity of radiographic methods, and abdominal and transabdominal ultrasound in detecting ossification centers in the fetal skeleton. These authors found the earliest visualization of ossification centers to be achieved by X-ray examinations. Using transvaginal ultrasound, these primary ossification centers were visible at the same time or 1 week thereafter. The delayed visualization of primary ossification centers, i.e., 1 or 2 weeks thereafter was achieved using abdominal ultrasound. As regards detecting skeletal dysplasias, a greater diagnostic precision was demonstrated using 3D-CT compared to 2D-US [4, 19]. Computed tomography allows observation of an examined structure in every plane and at any time, without sacrificing image detail after the examination. Besides, compared to 2D X-ray images, CT eliminates the overlap of anatomical structures and allows us to discern different body tissues. A factor that limits the applicability of CT examinations is a lack of numerical data referring to the fetal skeletal system at the specific weeks of pregnancy in comparison with routine ultrasound examinations. Currently, magnetic resonance imaging has become a clinical complement for ultrasound and is the best diagnostic tool used to assess fetal anatomy in both prenatal and post-mortem examinations. MRI is critically required in the second and third trimesters of pregnancy when ultrasound imaging offers either ambiguous or limited findings [7]. The use of MRI in fetal examinations refers mainly to congenital defects of the central nervous and skeletal systems and to congenital defects of thoracic and abdominal viscera [1]. Innovative cine-MRI techniques allow a multifaceted insight into the entire in utero fetus [18].

The main limitation of this study has been a relatively narrow fetal age, ranging from week 18, and a relatively small number of individuals, consisting of 42 human fetuses.

## Conclusions

1. Neither male–female nor right–left differences are found for any of the morphometric parameters of the ischium’s primary ossification center.
2. With relation to fetal ages in weeks, the ischium’s primary ossification center grows proportionately in vertical and sagittal diameters, second-degree polynomially in projection surface area, and logarithmically in volume.
3. The quantitative findings of the ischium’s primary ossification center are considered age-specific reference data of relevance in the diagnostics of innate defects.
